# Critical care capacity in Canada: results of a national cross-sectional study

**DOI:** 10.1186/s13054-015-0852-6

**Published:** 2015-04-01

**Authors:** Robert A Fowler, Philip Abdelmalik, Gordon Wood, Denise Foster, Noel Gibney, Natalie Bandrauk, Alexis F Turgeon, François Lamontagne, Anand Kumar, Ryan Zarychanski, Rob Green, Sean M Bagshaw, Henry T Stelfox, Ryan Foster, Peter Dodek, Susan Shaw, John Granton, Bernard Lawless, Andrea Hill, Louise Rose, Neill K Adhikari, Damon C Scales, Deborah J Cook, John C Marshall, Claudio Martin, Philippe Jouvet, on behalf of the Canadian Critical Care Trials Group and The Canadian ICU Capacity Group

**Affiliations:** Interdepartmental Division of Critical Care Medicine and Department of Medicine, University of Toronto, Toronto, Canada; Department of Critical Care Medicine, Sunnybrook Hospital, 2075 Bayview Avenue, Room D138, Toronto, Ontario M4N 3M5 Canada; Office of Situational Awareness and Operations, Centre for Emergency Preparedness & Response, Health Security Infrastructure Branch, Public Health Agency of Canada, 100 Colonnade Road, Ottawa, Ontario K1A 0K9 Canada; Adult Intensive Care, Island Health Authority, Royal Jubilee Hospital, 1952 Bay St, Victoria, BC V8R 1J8 Canada; Critical Care Research, Vancouver General Hospital, JPPN, ICU, Room 2469 899 12th Avenue West, Vancouver, BC V5Z 1M9 Canada; Division of Critical Care Medicine, 3C1.16 University of Alberta Hospital, 8440-112 Street, Edmonton, AB T6G 2B7 Canada; Memorial University of Newfoundland, Discipline of Medicine, Memorial University of Newfoundland, St John’s, NL A1B 3V6 Canada; Department of Anesthesia and Critical Care Medicine, and Population Health and Optimal Health Practices Research Unit, Centre Hospitalier Universitaire (CHU) de Québec and Université Laval, 1401, 18e Rue, H-037, Québec, G1J 1Z4 Canada; Centre Hospitalier Universitaire de Sherbrooke Service de médecine interne (local 4223), 3001 12e Av. N., Sherbrooke, Québec, J1H 5N4 Canada; Section of Critical Care Medicine and Section of Infectious Diseases, Departments of Medicine and Medical Microbiology, University of Manitoba, 710 Park Blvd, South Winnipeg, MB R3P-0X1 Canada; University of Manitoba, ON2051 – 675 McDermot Ave, Winnipeg, Manitoba R3E 0V9 Canada; Dalhousie University Medical Director, Trauma Nova Scotia, Halifax, Canada; Division of Critical Care Medicine, Department of Emergency Medicine, Room 349 Bethune Bld, 1276 South Park St, Halifax, NS B3H 2Y9, Canada; Division of Critical Care Medicine, Faculty of Medicine and Dentistry, University of Alberta, 2-124E Clinical Sciences Building, 8440-112 ST NW, Edmonton, T6G2B7 Canada; Department of Critical Care Medicine, Institute for Public Health, University of Calgary and Alberta Health Services, 3280 Hospital Drive NW, Calgary, AB T2N 4Z6 Canada; IH Critical Care Network Medical Director, Critical Care, Kelowna General Hospital, 2268 Pandosy St, Kelowna, BC V1Y 1T2 Canada; Critical Care Medicine, University of British Columbia. Vancouver Costal Health Research Institute, Research Scientist, Center for Health Evaluation and Outcome Sciences, 1081 Burrard Street, Vancouver, BC V6Z 1Y6 Canada; University of Saskatchewan College of Medicine’s Department of Anesthesiology, Perioperative Medicine and Pain Management, Royal University Hospital, 103 Hospital Drive, Saskatoon, Saskatchewan S7N 0W8 Canada; Medicine, Faculty of Medicine, University of Toronto. Head, Division of Respirology University Health Network, Mount Sinai Hospital, Women’s College Hospital, University Health Network, 11-124 PMB, Toronto General Hospital, 585 University Ave, Toronto, Ontario M5G 2N2 Canada; Department of Surgery, University of Toronto. Provincial Lead, Critical Care and Trauma, Critical Care Services Ontario, St. Michael’s Hospital 30 Bond Street, Toronto, ON M5B 1W8 Canada; Lawrence S. Bloomberg Faculty of Nursing, University of Toronto, Sunnybrook Hospital, 2075 Bayview Avenue, Room D138, Toronto, Ontario M4N 3M5 Canada; St. Joseph’s Hospital, McMaster University, Hamilton, Ontario L8N 3Z5 Canada; Keena Research Center of the Li ka Shing Knowledge Institute of St. Michael’s Hospital, 30 Bond Street, Toronto, ON M5B 1W8 Canada; Critical Care Western and London Hospitals. Professor, Schulich School of Medicine & Dentistry, Western University Scientist, Centre for Critical Illness Research, Lawson Health Research Institute, Room D2-528 London Health Sciences Centre – Victoria Hospital PO Box 5010 800 Commissioners Road, East London, ON N6A 5W9 Canada; Director of the Pediatric Intensive Care Unit/chef de service des soins intensifs pédiatriques, CHU Sainte-Justine, 3175 Chemin de Côte Sainte Catherine, Montreal, Québec H3T 1C5 Canada

## Abstract

**Introduction:**

Intensive Care Units (ICUs) provide life-supporting treatment; however, resources are limited, so demand may exceed supply in the event of pandemics, environmental disasters, or in the context of an aging population. We hypothesized that comprehensive national data on ICU resources would permit a better understanding of regional differences in system capacity.

**Methods:**

After the 2009–2010 Influenza A (H1N1) pandemic, the Canadian Critical Care Trials Group surveyed all acute care hospitals in Canada to assess ICU capacity. Using a structured survey tool administered to physicians, respiratory therapists and nurses, we determined the number of ICU beds, ventilators, and the ability to provide specialized support for respiratory failure.

**Results:**

We identified 286 hospitals with 3170 ICU beds and 4982 mechanical ventilators for critically ill patients. Twenty-two hospitals had an ICU that routinely cared for children; 15 had dedicated pediatric ICUs. Per 100,000 population, there was substantial variability in provincial capacity, with a mean of 0.9 hospitals with ICUs (provincial range 0.4-2.8), 10 ICU beds capable of providing mechanical ventilation (provincial range 6–19), and 15 invasive mechanical ventilators (provincial range 10–24). There was only moderate correlation between ventilation capacity and population size (coefficient of determination (R^2^) = 0.771).

**Conclusion:**

ICU resources vary widely across Canadian provinces, and during times of increased demand, may result in geographic differences in the ability to care for critically ill patients. These results highlight the need to evolve inter-jurisdictional resource sharing during periods of substantial increase in demand, and provide background data for the development of appropriate critical care capacity benchmarks.

**Electronic supplementary material:**

The online version of this article (doi:10.1186/s13054-015-0852-6) contains supplementary material, which is available to authorized users.

## Introduction

ICUs provide life-supporting treatments to critically ill patients. ICU resources are limited and costly in Canadian hospitals. Clinicians must consider the possible benefits of admission to ICU, and hospital administrators must coordinate the provision of procedures and surgeries requiring critical care with existing capacity [1,2]. During periods of increased demand for ICU resources, such as during infectious outbreaks or pandemics, it can be difficult to match available resources to clinical demands [3-5], resulting in the potential for rationing of ICU care [6].

There is substantial global variation in the capacity to provide critical care [7]. Previous estimates using national health administrative data indicate that Canada has far fewer ICU beds per capita than the United States, but similar numbers of ICU beds to those in many Western European nations [8]. Because healthcare is a provincial portfolio in Canada, differences in provincial priorities may translate to differences in availability of specific resources. Moreover, because critical care services represent one of the most expensive components of the healthcare system [9], cost is an additional reason for regional differences.

Although there have been prior provincial assessments [10-12], our overall national critical care capacity is unknown. Therefore, during the period after the 2009 to 2010 influenza A (H1N1) pandemic, the Canadian Critical Care Trials Group undertook a national survey of all acute care hospitals to determine the number of critical care beds and mechanical ventilators, as well as the availability of specialized support for respiratory failure in critically ill adults and children. We hypothesized that comprehensive national data on ICU resources would permit a better understanding of regional differences in system capacity in Canada, inform the potential need for inter-jurisdictional resource sharing during periods of increased national and provide background data for the development of appropriate critical care capacity benchmarks.

## Methods

### Questionnaire development

We developed a capacity survey to tabulate each hospital’s critical care resources using rigorous methodology [13]. Questionnaire development consisted of item generation and reduction with input from critical care physicians, nurses, respiratory therapists, clinical researchers, and research ethics officers. The questionnaire was subsequently tested for sensibility and reliability [13]. Final domains included hospital and respondent characteristics, ICU type, ICU beds, invasive ventilators, availability of specialized supports for respiratory failure, and a field for qualitative comments. Recognizing no uniformly accepted definition, we used a sensitive definition of ICU and subsequently determined capacity for ongoing invasive mechanical ventilation in an ICU (that is, not the emergency department or post-anesthetic care unit) (see Additional file 1).

### Respondents and survey completion

Canadian Critical Care Trials Group members in each province were enlisted as local collaborators and site champions in order to identify acute care hospitals and ICUs through snowball sampling of colleagues and relevant government-specific and hospital-specific contacts. In some provinces, pre-existing lists had been generated by ministries or departments of health (British Columbia, Alberta, Ontario, New Brunswick) or critical care societies (Quebec). The search for acute care institutions was supplemented by a manual search of the *Guide to Canadian Healthcare Facilities* [14], pre-existing hospital lists of the Public Health Agency of Canada, Google Maps, and web searches for all hospitals.

An ICU physician lead for each hospital was identified and contacted by email or telephone. Subsequently, nursing and respiratory therapy leaders were contacted by email or telephone from pre-existing lists, referral from nearby institutions, or through telephone solicitation directly to the hospital or ICU. The survey was then transmitted by email or fax for self-administration, or administered via telephone to physicians, nurses, and/or respiratory therapists. In hospitals with more than one ICU, additional ICU-specific contacts were established by subsequent telephone or email communication. In rare cases where no ICU-specific contact could be identified, or the leader did not respond (generally for small hospitals that had no geographically distinct ICU), we obtained information from an informed clinician who was primarily affiliated with another nearby hospital.

Respondents were to provide capacity data at the time of the survey. We attempted to complete data collection for each site within a 3-month time frame to limit temporal changes in any measure. In some cases, there was uncertainty about the number of available ICU beds because of staffing shortages. We asked respondents to answer based upon how many ICU beds were usually available for admission should staffing not be a limitation. We attempted to resolve uncertainty about the number of ICUs and bed numbers capable of ventilation through communication with more than one local physician and nurse leader. Respiratory therapy leaders resolved uncertainty about numbers of ventilators, and availability of inhaled nitric oxide, high-frequency oscillatory ventilation, and extracorporeal membrane oxygenation.

### Analyses

Descriptive statistics are presented as counts, proportions, medians (with range or interquartile range), and means (± standard deviation) as appropriate using Microsoft Excel (Redmond, WA, USA) or SAS (Carey, NC, USA). We tested the relationship between the number of ICU beds capable of ventilation and the population by health and census regions using the *R*^2^ coefficient of determination. Canadian and provincial population census numbers in 2009 to 2010 were derived from Statistics Canada census projections [15]. Spatial analyses were conducted in collaboration with the Public Health Agency of Canada in order to map the locations of hospitals and facilities across Canada that had ICU beds with capacity for invasive ventilation. Moreover, the population within a 40 km service area of each of these hospitals was identified using 2006 census data [16]. Network analyses using both health region and census division service were performed using CanMap route logistics for road networks and enhanced points of interest [17], hydrology data from Statistics Canada [18], and aerial imagery using Bing™ maps aerialand ArcGIS 10.0 (ArcView) with the NetworkAnalyst extension from ESRIC (see Additional file 1) [19].

### Confidentiality and ethics

Research ethics approval for this study was granted by Sunnybrook Hospital in April 2009 without the need for consent because only institutional data and no individual patient-related data were collected. Study funders and provincial governmental officials who provided data had no role in its analysis or publication.

## Results

### Overall resources

We identified 362 acute care centers in 10 provinces and three territories. There were 286 hospitals with at least one self-designated ICU that was capable of providing invasive mechanical ventilation. ICUs in 22 hospitals had designated beds for pediatric patients, and 15 hospitals had stand-alone pediatric ICUs. The vast majority of ICUs (280, 97.9%) were capable of caring for mixed, medical and surgical, patient populations. Many ICUs (27, 9.4%) cared for medical, cardiac, or cardiothoracic surgery patients either in isolation or in addition to other critically ill patients. Two ICUs cared exclusively for critically burned patients, whereas four routinely cared for some burned patients, and three ICUs cared exclusively for neurologically injured or neurosurgical patients.

In hospitals with an ICU capable of providing invasive mechanical ventilation there was a total of 4,309 beds, of which 3,170 were specifically designated for mechanical ventilation. Among hospitals with an ICU capable of providing invasive mechanical ventilation there was a median of nine (interquartile range (IQR) 5 to 18) ICU beds with eight (IQR 3 to 14) beds available for patients who required invasive mechanical ventilation. There was a total of 4,982 ventilators capable of providing invasive mechanical ventilation, including ventilators used for patient transportation (but not those primarily used to deliver inhalational anesthesia during surgery) and a median of 10 (IQR 5 to 23) invasive ventilators per hospital (Table 1). In terms of specialized support for respiratory failure, there were 178 high-frequency oscillatory ventilators in 72 (25.2%) hospitals that had an ICU capable of delivering ventilation, inhaled nitric oxide in 79 (27.6%) hospitals, and extracorporeal membrane oxygenation in 39 (13.6%) hospitals. All three supports were available in 32 (11.1%) hospitals – all being university teaching centers.

**Table 1:** **Number of ICUs and ventilation and oxygenation capacity across provinces**

**Region**	**Hospitals with ICUs with ventilation capacity**	**ICU beds capable of invasive ventilation**	**Ventilators capable of invasive ventilation**	**High-frequency oscillatory ventilators**	**Hospitals with ICUs with iNO**	**Hospitals with ICUs with ECMO**
Newfoundland	14	98	124	7	3	2
Nova Scotia	14	141	191	6	7	2
Prince Edward Island	2	18	22	0	0	0
New Brunswick	9	103	113	1	3	0
Quebec	87	885	1,197	40	25	12
Ontario	84	1,122	2,101	65	19	11
Saskatchewan	13	108	235	10	3	3
Manitoba	10	93	151	10	3	2
Alberta	16	292	373	18	8	3
British Columbia	34	304	460	21	7	4
Territories	3	6	15	0	1	0
Canada	286	3,170	4,982	178	79	39

ECMO, extracorporeal membrane oxygenation; iNO, inhaled nitric oxide.

### Resources across provinces and territories

**Figure 1:**
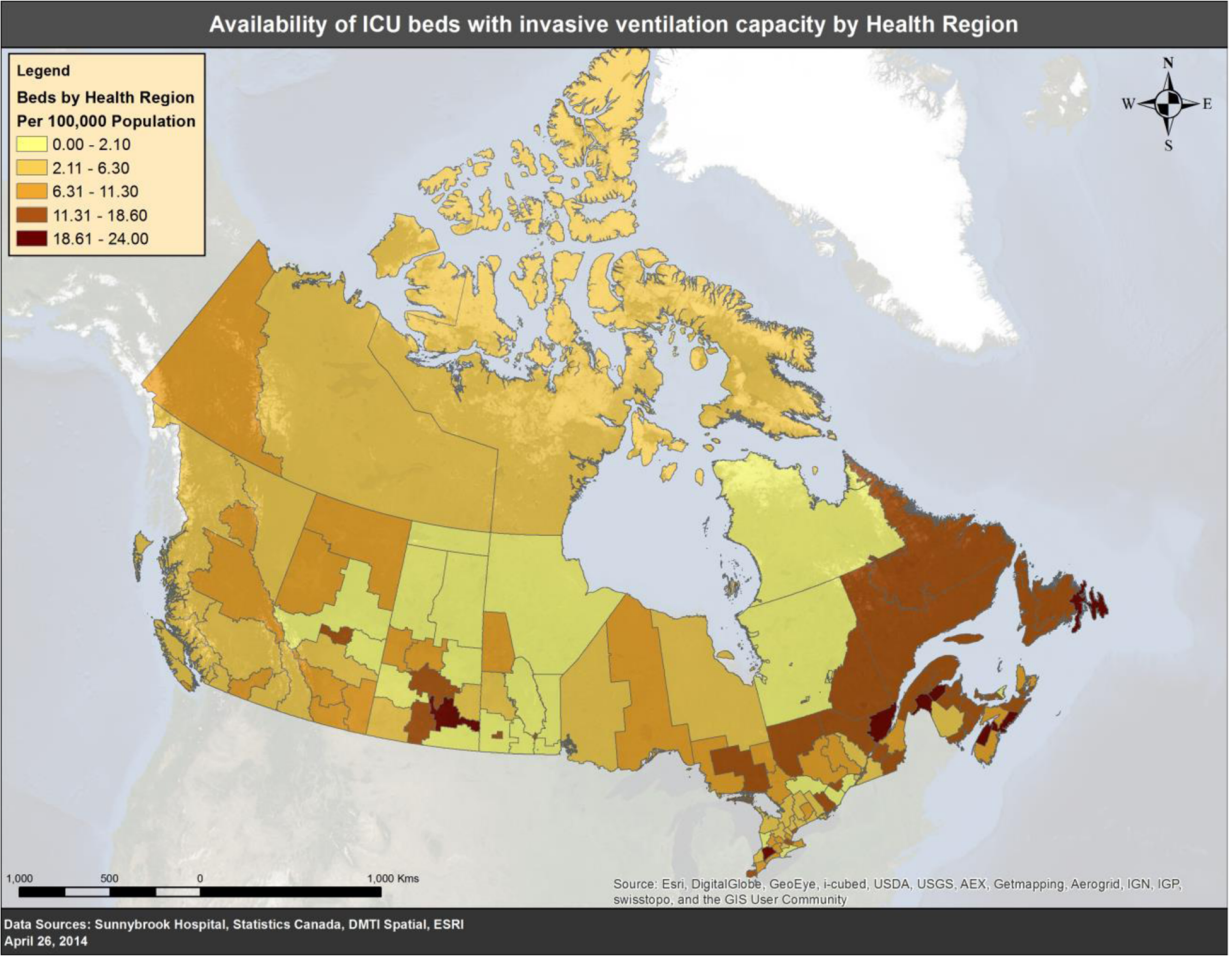
**ICU beds capable of invasive mechanical ventilation per 100,000 Canadian population according to health region.**

There was substantial variation in capacity to provide critical care among provinces and territories. The median number of ICUs per province was 14 (IQR 10 to 25). The median number of ICU beds per province was 122 (IQR 110 to 337), and the median number available for mechanical ventilation was 108 (IQR 97 to 298). The median number of mechanical ventilators per province was 201 (IQR 131 to 435). Per 100,000 population in 2009 and 2010, there were 0.9 hospitals with ICUs capable of providing mechanical ventilation (range 0.4to 2.8), 10 ICU beds capable of providing mechanical ventilation (range 6 to 19), and 15 invasive mechanical ventilators (range 10 to 24) (Figure 1, Table 2; Figure S1A in Additional file 1).

**Table 2:** **ICUs and ventilation capacity according to 2009 population across provinces**

**Region**	**Hospitals with ICUs per 100,000 population (%)**	**ICU beds with ventilation capacity per 100,000 population (%)**	**Invasive ventilators per 100,000 population (%)**
Newfoundland	2.8	19.3	24.4
Nova Scotia	1.5	15.0	20.3
Prince Edward Island	1.4	12.8	15.6
New Brunswick	1.2	13.8	15.1
Quebec	1.1	11.3	15.3
Ontario	0.6	8.6	16.1
Saskatchewan	1.3	10.5	22.8
Manitoba	1.0	9.0	14.7
Alberta	0.4	7.9	10.1
British Columbia	0.8	6.8	10.3
Territories	2.7	5.5	13.7
Canada	0.9	9.5	14.9

### Resources according to the type of hospital

There was considerable concentration of ICU resources for mechanical ventilation in teaching hospitals as compared with community hospitals. The total number of teaching and community ICU beds capable of mechanical ventilation was 2,027 and 1,143, respectively, with a median number of 20 (IQR 11 to 31) beds and five (IQR 2 to 8) beds, respectively (*P* <0.001). There was moderate correlation between the number of ICU beds capable of mechanical ventilation and the population size for both health region divisions (*R*^2^ = 0.771) and census divisions (*R*^2^ = 0.809) (Additional file 1).

### Other qualitative characteristics of ICUs

Complementary qualitative data acquired in our survey shed important insights into critical care capacity in Canada. Some hospitals with small ICUs (that is, fewer than six ICU beds) commented that despite their capacity for ongoing ventilation, it was common practice to attempt to transfer patients to larger ICUs after a variable period of time (many days to a week). Many hospitals commented that lack of personnel (especially nurses) prevented the full utilization of ICU beds and ventilator capacity. In hospitals with more than one ICU, conflicts in admission decision processes prevented optimal ICU bed utilization. For instance, open beds were maintained in subspecialty ICUs while general ICUs were at or above capacity.

## Discussion

We found substantial variation in the numbers of ICU beds, as well as the capacity for mechanical ventilation and specialized support for respiratory failure among ICUs in Canada. These findings were not fully explained by the size of the population. This variation in capacity may result in differential decision-making about who can receive ICU support, and which services can be supported in specific hospitals and regions during times of increased demand [3,5].

Prior work by our group using health administrative data from the Canadian Institute for Health Information estimated that there were 319 ICUs, 3,388 total adult ICU beds (representing 3.4% of all acute care hospital beds), and 13.5 ICU beds per 100,000 population [8]. However, these data were based on a more liberal definition of critical care beds, did not include data from Quebec, did not include any interprovincial comparisons, did not estimate the capacity to treat critically ill patients requiring mechanical ventilation, and were generated one decade ago. Our assessment of ICU beds per 100,000 population places Canada near the median of high-income and Organization for Economic Co-operation and Development countries, notably above the United Kingdom but well below the United States, Germany, and Belgium [8,7,20,21,22].

Without knowledge of Canadian critical care capacity, and in the absence of provincial, national, or international targets for population-based critical care resources, there has been limited national attention to ensuring optimal distribution among regions. The results of this survey highlight expected north–south geography-based capacity trends, but also an unanticipated apparent east–west gradient with relative increased capacity among the Atlantic Provinces, in comparison with central and western Canada. Some members of our group have previously reported wide variation in ICU capacity within British Columbia and an inverse relationship between ICU beds, population density, and population growth, highlighting the potential for mismatch in demand and capacity in Canada [11].

Some variation in distribution of healthcare services is probably a consequence of differential regional models of healthcare delivery. For example, there are substantial differences in population density across Canada, with marked north-to-south increases in density – approximately 80% of Canadians live within 160 km of the Canada–United States border [23] – and there is marked intra-provincial urban–rural variability [24]. Many systems of intra-provincial regionalization of care that are responsive to population density, geographic barriers, or evolved regional care systems also may lead to differences in the distribution of critical care services. Moreover, despite provincial administration of most healthcare services, there is also some well-established inter-provincial ICU care, with regionalized trauma services, specialized care delivery for northern territories among bordering provinces, and populations of one province that are closest to a specialized healthcare center in a neighboring province. It is not clear, however, whether specialized services’ referral relationships work well during times of healthcare crisis such a pandemic when regions may react to future uncertainty by trying to conserve resources for more local use.

This study has important limitations. First, this survey was carried out using existing national and provincial databases of hospitals, and it is possible that some acute care hospitals may have been missed. However, we subsequently employed snowball sampling and web and map searching techniques to identify all hospitals and ICUs in each province, and then sought out a combination of physician, nurse, respiratory therapist, and hospital administrator leaders to derive current ICU beds and ventilator capacity at each hospital. After compiling local data, each participant and provincial health authority was given the opportunity to critique the aggregate estimates to improve accuracy. Second, population denominator-based comparisons may not be the optimal mechanism for normalization in all regions with varying population density, age demographic differences, geographic barriers, and distinct systems of regionalized care for some tertiary and quaternary services such as trauma and transplantation. However, our results indicate relatively wide variability in ICU capacity among provinces and therefore may provide helpful inter-provincial comparisons. Third, this study focused on a very narrow spectrum of services needed to provide critical care – ICUs, beds, ventilators, and specialized supports for respiratory failure. It was beyond the scope of this survey to evaluate personnel (dieticians, nurses, pharmacists, physicians, physiotherapists, respiratory therapists, social workers) or other resources that are essential to the care of critically ill patients. Indeed, lack of available critical care clinical staff is among the most common reason for limitations in bed availability [25-27]. Future resource planning must address this key knowledge gap. Fourth, ICU resources are not static, and this survey represents a period prevalence of approximately 3 months at the hospital level and approximately 1 year among all sites, in a period after the H1N1 pandemic where knowledge of ICU capacity may have been greatest.

Our results highlight the need to examine capacity both in relation to local needs and in comparison with other regions. It is important to note that the organization of critical care within Canada has not been static since conducting this survey. Alberta has reorganized critical care services under one structure, with a standardized provincial bedside clinical information system/electronic medical record [28]. Since the severe acute respiratory syndrome experience, the Ministry of Health and Long-term Care in Ontario has maintained a Critical Care Strategy to oversee a similar cataloguing of critical care services including twice-daily clinical updates of every patient in ICUs into a centralized electronic database that facilitates critical care inter-facility transportation services, reporting on quality metrics and decision-making on surge capacity [29]. British Columbia and Nova Scotia have recently formed Critical Care Working Groups within the Ministry of Health to coordinate data collection and reporting, improvement of care processes, transportation of critically ill patients, and improvement of staffing models in ICUs. In 2011 Quebec created a Groupe d’Experts en Soins Intensifs working with the Ministry of Health to improve quality and accessibility of ICUs and a mandate to establish provincial ICU capacity.

One of the lessons learned from the severe acute respiratory syndrome and influenza A (H1N1) pandemics is that infectious outbreaks do not respect regional health boundaries [30,31] and that individual regions may be clinically overwhelmed while others are unaffected. Of relevance to surge planning, we were able to quantify the excess numbers of invasive mechanical ventilators relative to ICU beds, highlighting capacity that may exist beyond existing ICU beds. The ability to provide advanced oxygenation with one of three modes of support was available in a minority of hospitals. Furthermore, this expertise was unevenly distributed across provinces, and was focused at university-affiliated teaching hospitals. However, we were unable to gauge experience with specialized ventilation alongside capacity. We did not determine capacity for other techniques such as prone ventilation, which may be less dependent upon specific technology, more dependent upon generation of a local experience base, and have a greater evidence base for efficacy than either early high-frequency ventilation or use of inhaled nitric oxide [32]. This variable and uneven distribution of expertise observed in this study demands that we evolve a system in which excess capacity in one region may aid another, either through safe transportation of patients or short-term movement of equipment or personnel to existing or temporary facilities [33].

## Conclusion

ICU resources vary widely across Canadian provinces, and during times of increased demand may result in geographic differences in the ability to care for critically ill patients. These results may guide future decision-making, but must also be complemented by estimates of current and future healthcare personnel supply and projections of demand for critical care from an aging population [34]. Greater ongoing knowledge of regional critical care resources may help us respond to increases in demand from unpredictable events such as infectious outbreaks or regional medical emergencies. While some regional imbalances may persist, there should be deliberate planning for mechanisms to deal with both unexpected and day-to-day surge in demand. Mechanisms are needed to rapidly share and deploy resources – both equipment and personnel – across provincial boundaries to deliver a more equitable, coordinated, and responsive system of healthcare for critically ill Canadians.

## Key messages

- We identified all ICU beds and mechanical ventilators for critically ill patients in Canada.
- There was substantial variability in inter-provincial capacity.
- There was only moderate correlation between ventilation capacity and population size.
- During times of increased demand, variability in ICU resources may result in geographic differences in the ability to care for critically ill patients.
- These results highlight the need to evolve inter-jurisdictional resource, and provide background data for the development of appropriate critical care capacity benchmarks.

## Additional file

Additional file 1:
**Contains Figure S1A showing ICU beds capable of invasive mechanical ventilation per 100,000 Canadian population according to census division, Figure S2 showing the relationship between number of ICU beds capable of ventilation and population by health region, and Figure S3 showing the relationship between number of ICU beds capable of ventilation and population by census division, and contains the mapping methodology technical notes and the Canadian Critical Care Trials Group Capacity Survey.**

## Abbreviation

IQR: interquartile range
