# Supplementary material for: Critical care capacity in Canada: results of a national cross-sectional study
Source: Crit Care. 2015 Apr 1;19(1):133. doi: 10.1186/s13054-015-0852-6 (PMC4426537; doi:10.1186/s13054-015-0852-6)
Supplement: Additional file 1: — Contains Figure S1A showing ICU beds capable of invasive mechanical ventilation per 100,000 Canadian population according to census division, Figure S2 showing the relationship between number of ICU beds capable of ventilation and population by health region, and Figure S3 showing the relationship between number of ICU beds capable of ventilation and population by census division, and contains the mapping methodology technical notes and the Canadian Critical Care Trials Group Capacity Survey. [file 13054_2015_852_MOESM1_ESM.pdf]

Title: Critical Care Capacity in Canada During the Influenza A(H1N1) Pandemic of 2009-10: Results of a National Survey

Additional file 1

Figure 1A: ICU Beds Capable of Invasive Mechanical Ventilation per 100,000 Canadian Population According to Census Division.

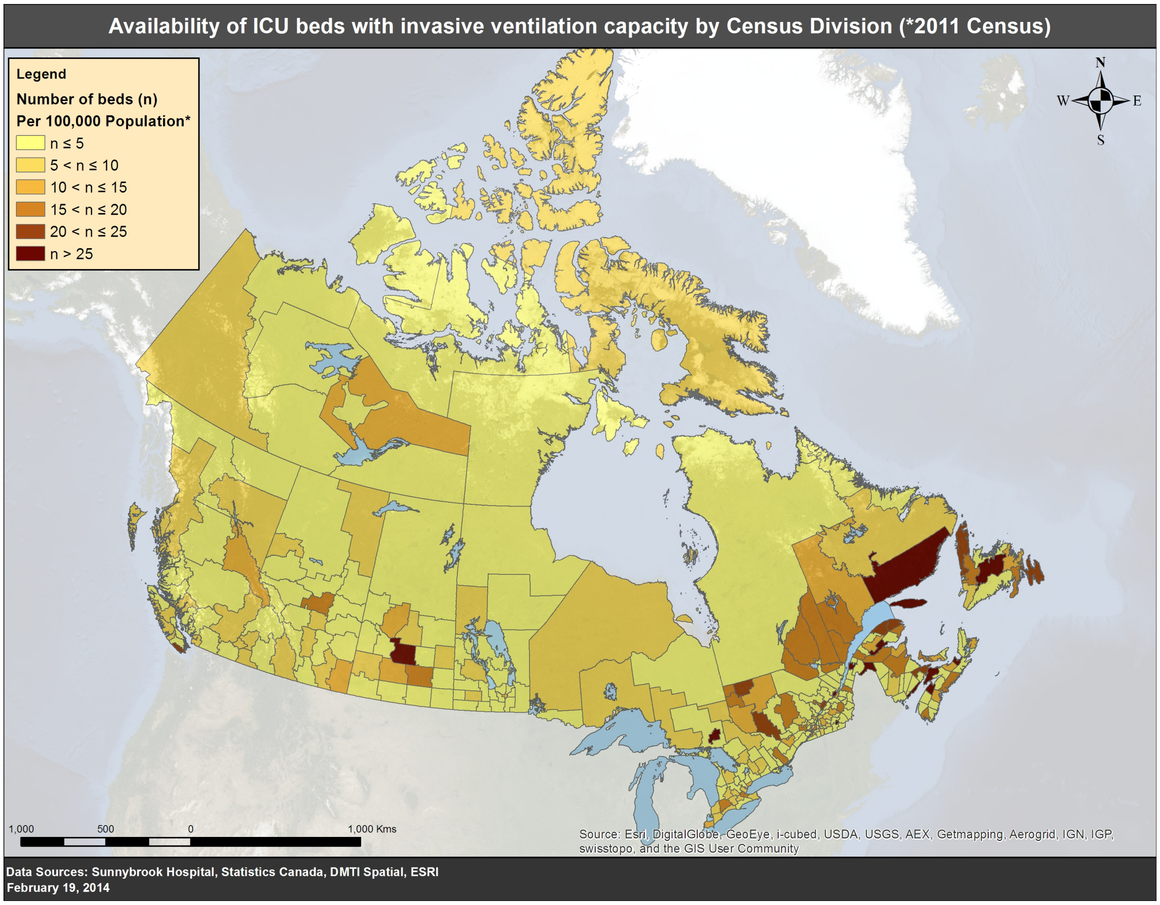

Title: Critical Care Capacity in Canada During the Influenza A(H1N1) Pandemic of 2009-10: Results of a National Survey

Figure 2. Relationship Between Number of ICU Beds Capable of Ventilation and Population by Health Region.

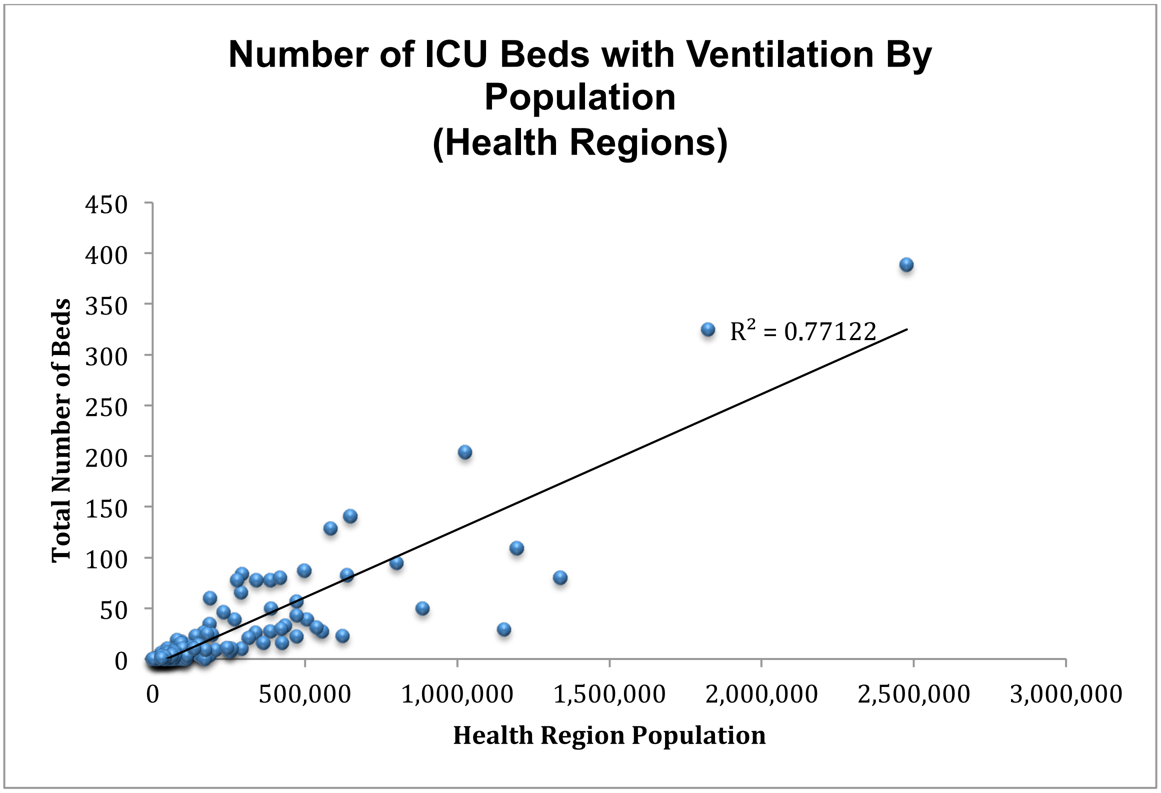

Title: Critical Care Capacity in Canada During the Influenza A(H1N1) Pandemic of 2009-10: Results of a National Survey

Figure 3. Relationship Between Number of ICU Beds Capable of Ventilation and Population by Census Division.

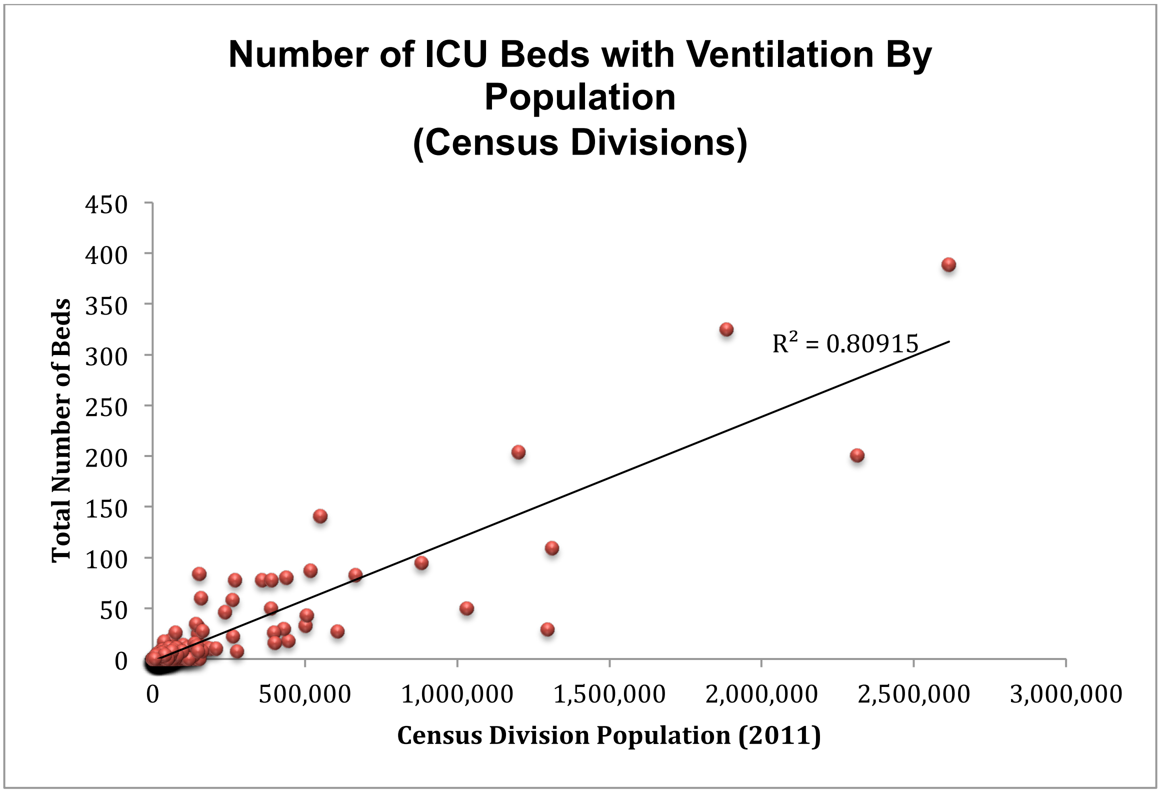

Title: Critical Care Capacity in Canada During the Influenza A(H1N1) Pandemic of 2009-10: Results of a National Survey

Mapping Methodology Technical Notes

DATA SOURCES & SOFTWARE

Client data:
- Hospital names and the associated attributes of interest (e.g. numbers of beds, etc) were provided by Dr. Fowler
- Linking of hospitals with location identifiers was completed by Corey Oliver and the final file was delivered to Philip AbdelMalik and Kara Hayne

- Spatial data (purchased and held by the Office of Public Health Practice, Public Health Agency of Canada):
  - CanMap Route Logistics version 2010.3 for road networks: DMTI Spatial, http://www.dmtispatial.com
  - Enhanced Points of Interest version 2010.3 for hospital locations: DMTI Spatial, http://www.dmtispatial.com
  - Hydrology: Statistics Canada, http://www.statcan.gc.ca
  - Aerial imagery: BingTM maps aerial (http://www.bing.com/maps) directly in GIS software (see Software)
- Census data (purchased and held by the Office of Public Health Practice, Public Health Agency of Canada):
  - 2006 census data at the dissemination area (DA) level: Statistics Canada, http://www.statcan.gc.ca

- Software
- ArcGIS 10.0 (ArcView license) from ESRI® (http://www.esri.com)
- Network Analyst extension for ArcGIS 10.0 (http://www.esri.com)
- Microsoft Office Excel 2003 (http://www.microsoft.com)
- Microsoft Office Word 2003 (http://www.microsoft.com)

DATA CLEANUP

1. Initial join attempt matched 340 out of 366 hospital records (92.9% success rate)

2. Blank spaces found after the Points of Interest (POI) ID entries associated with the hospitals in some records – these were all removed

3. Multiple POIs for six records (due to multiple facilities). Queries were resolved.

   a) A new field labeled "LINK" was created in the dataset to allow for identification of "linked" (combined) facilities.
   b) For each of the records above, duplicate records were added as appropriate (i.e. one duplicate record for all except for the Victoria General *et al* records, for which two duplicate records were added to capture all three institutions).
   c) All non-duplicated records were assigned a **unique** LINK value; duplicated records were assigned the **same** LINK value
   d) New number of records, n=373

4. Missing Points of Interest were queried and resolved.

GENERATING A SHAPEFILE FROM THE ICU DATA

1. The data was opened and cleaned in Microsoft Excel: columns were re-named; extra spaces were removed and missing POI_IDs were added to the original data
2. The ICU data was imported into ArcGIS 10, as was the Enhanced Points of Interest (EPOI) Dataset, version 2010.3
3. The EPOI dataset was joined to the ICU dataset (target dataset) on the POI_ID field. All 373 records in the target dataset were retained
4. The joined data was exported as a CSV file, and missing latitudes and longitudes were entered as given above using MS Excel.
5. The complete ICU dataset was re-imported into ArcGIS and a shapefile was generated using the latitudes and longitudes. The resulting map is provided in the accompanying **MAPS** document.

NETWORK ANALYSIS - SERVICE AREA

The road network for all of Canada was added to the project using the CanMAP Route Logistics data product version 2010.3.2.A

Title: Critical Care Capacity in Canada During the Influenza A(H1N1) Pandemic of 2009-10: Results of a National Survey

Canadian Critical Care Trials Group Capacity Survey

Canadian Critical Care Trials Group ICU Capacity Survey

The Canadian Critical Care Trials Group is **asking for your help in determining our collective capacity for caring for critically ill patients** in hospitals across Canada.

Attached is a short survey that aims to determine the current capacity to care for critically ill patients at your hospital.

There will be many local distinctions about what constitutes an Intensive Care Unit ("ICU") and an "ICU bed". For our purposes, **ICU refers to a geographic place where patients receive specialized nursing and medical care with an increased Nurse:Patient ratio in comparison to the general ward**. In some ICUs, but not all, there can be continuous monitoring of vital signs, heart rhythm and oxygen saturation, provision of invasive and non-invasive ventilation, and intravenous vasoactive medication. Except where designated as "Pediatric", ICUs refer to locations with capacity to care for adult patients.

We believe **this knowledge is critically important, especially so as we head into our Influenza season** with the possibility of increased number of cases of critically ill patients. Knowledge of capacity will be important for planning for resources for patients who may need intensive care.

Our aim is to have these surveys completed locally in a very short time-frame and to **deliver the results back to individual sites, hospitals, health care policy makers at local, provincial and national levels** in order that we better understand capacity and ability provide critical care to patients over this coming year.

We also commit to submitting these results for publication in a medical journal and we will list all collaborators under a single authorship of the Canadian Critical Care Trials Group.

Thank you for your help in filling out this survey for your hospital!

Sincerely, on behalf of the CCCTG

Title: Critical Care Capacity in Canada During the Influenza A(H1N1) Pandemic of 2009-10: Results of a National Survey

Canadian Critical Care Trials Group ICU Capacity Survey

A. Site

1. Hospital Name: ___________________________

2. Town/City: ______________________________

3. Province: _______________________________

B. Contact Information

1. Name: __________________________________

2. Phone: (_____)___________________________

3. Fax: (_____)___________________________

4. Email address: ___________________________

5. Profession: MD, RN, RT, Research Coordinator, Other: ______________

C. Does your hospital have an areas designated as an Intensive Care Unit or Critical Care Unit (can refer to the above definition if clarification is required)?

Yes / No

If Yes, please fill out the following information (pages 3 and 4

Title: Critical Care Capacity in Canada During the Influenza A(H1N1) Pandemic of 2009-10: Results of a National Survey

D. Intensive Care Unit Capacity

Type of ICU	Present in your Hospital? (circle)	Total Number of ICU Beds	Total Number of ICU Beds with Invasive Ventilation Capability	Number of Ventilators Capable of Delivering Ongoing Invasive Mechanical Ventilation (*not* including HFO ventilators or those only used in operating room for anesthesia)	Number of Transport Ventilators (*not* including ventilators only used in operating room)	Total Number of Ventilators in Hospital with Capable of Invasive Ventilation (*not* including HFO ventilators or those only used in operating room for anesthesia)
Medical-Surgical	Yes / No					
Medical Only	Yes / No					
Surgical Only	Yes / No					
Neurosurgical	Yes / No					
Cardiac Surgery	Yes / No					
Trauma	Yes / No					
Medical Surgical Step-up	Yes / No					
Medical Only Step-Up	Yes / No					
Surgical Only Step-up	Yes / No					
Cardiac Care Unit	Yes / No					
Pediatric	Yes / No					
Other ICU 1	Yes / No					
Other ICU 2	Yes / No					

E. Intensive Care Unit Capacity, continued from last page

1. Total Number of **High Frequency Oscillatory Ventilators (HFO)** in any area of your hospital:

____

2. Can your hospital / ICU deliver **inhaled nitric oxide** to critically ill patients in an ICU?

(Yes / No)

3. Does your hospital have the ability to provide **extracorporeal membrane oxygenation (ECMO)** to critically ill patients in an ICU?

(Yes / No)

Other Comments Regarding Capacity at Your Hospital:
